# Prediction of Cervical Lymph Nodes Metastasis in Papillary Thyroid Carcinoma (PTC) Using Nodal Staging Score (NSS)

**DOI:** 10.1155/2022/9351911

**Published:** 2022-10-15

**Authors:** Jianliang Zhang, Guangwei Jia, Yang Su, Zhongji Zhang, Hui Xiong, Qiu Xu, Shan Meng

**Affiliations:** ^1^Thyroid and Breast Surgery, Nanyang First People's Hospital, Nanyang, Henan Province 473010, China; ^2^Department of Nuclear Medicine, Nanyang First People's Hospital, Nanyang, Henan Province 473010, China

## Abstract

**Background:**

Cervical lymph node metastasis is commonly seen in papillary thyroid carcinoma. Surgery is the preferred treatment for PTC with cervical lymph node metastasis. There is no alternate ultrasound, neck CT, and thyroglobulin (Tg) methods to assess the occult lymph node metastasis. For moderate-and high-risk PTC, the number of lymph nodes to be dissected should be increased to remove the occult lymph node metastasis.

**Objective:**

This study was designed to develop a nodal staging score model to predict the likelihood of lymph node metastasis in papillary thyroid carcinoma (PTC), and further guide the treatments. *Material and Methods.* Data were collected from the SEER database. Patients with PTC from 2000 to 2005 were selected. The beta-binomial model was adopted to establish a nodal staging score (NSS)-based model. The NSS-based model was built according to gender, age, extrathyroidal invasion, tumor multifocality, tumor size, and T stage of the patients. A total of 12,431 PTC patients were included in our study. Various types of lymph nodes were examined based on various categories (incidence, risk assessment) to evaluate the results.

**Results:**

5,959 (47.9%) patients in the study were positive and 6,472 (52.1%) were confirmed negative for lymph node metastasis. The corrected incidence of lymph node metastasis was higher than that of direct calculation, regardless of the factors that affected lymph node metastasis. There were significant differences in the OS of PTC patients among the four groups and T stage (*p* is less than 0.05), indicating that cervical lymph node metastasis would have an impact on the prognosis of patients.

**Conclusion:**

In conclusion, an NSS-based model base on a variety of clinicopathological factors can be used to predict lymph node metastasis. It is important to evaluate the risk of occult lymph node metastasis in the treatment of PTC.. Since, this statistical model can describe the risk of occult lymph node metastasis in patients; therefore, it can be used as basis for decision-making related to the number of lymph nodes that can be dissected in operations.

## 1. Introduction

Papillary thyroid carcinoma (PTC) is the most common pathological type of thyroid cancer [1–15]. Cervical lymph node metastasis is commonly seen in PTC and occurs in 20–50% of patients before the initial treatment [1–3]. Surgery is the preferred treatment for PTC with cervical lymph node metastasis. At present, the American Thyroid Association (ATA) recommends that preventive central lymph node dissection should be performed in the treatment of primary tumors for patients with moderate-and high-risk PTC, and cervical or mediastinal lymph node dissection should be performed after the metastasis is confirmed through puncture pathology [4]. Currently,ultrasound is recommended as the main means of preoperative lymph node evaluation [2, 5, 6]. However, for patients with no abnormal lymph nodes clinically before surgery, 77% of them still developed persistent disease of lymph nodes after receiving thyroid cancer surgery [7], requiring reoperation. Now there is no better method to assess occult lymph node metastasis other than ultrasound, neck CT, and thyroglobulin (Tg) [8]. So, lymph node metastasis can be detected by postoperative pathology. In the case of moderate-and high-risk PTC, the number of lymph nodes that are involved in dissection should be increased to remove the occult lymph node to the maximum extent [9]. But if this number is increased, an individual's life might be at risk. Therefore, an individualized plan for this purpose can increase the thoroughness of the procedure and additional trauma can be averted as much as possible. This study offers a quantitative and intuitive assessment method for the negative predictive value of lymph node examination. The prediction of metastasis and recurrence by the NSS model has not been carried out due to a data type limitation in the SEER database. In the SEER database, the metastatic areas have not been classified, so a further search cannot be carried out. Therefore, a perfect NSS model requires further research with a larger sample size and more comprehensive data.

## 2. Materials and Methods

### 2.1. Sample Collection

Sample data was collected from Surveillance, Epidemiology, and End Results (SEER), National Cancer Institute, USA [10]. A total of 12,431 patients with PTC (ICD-O-3 codes: 8050, 8260, 8340, 8341, 8343, and 8344) from 2000−2005 were selected for our study. The patients were divided into two groups according to their ages: (1) patients who were of age 45 or under 45 and (2) patients older than 45. Four groups according to tumor size included: (1) patients whose tumor sizes were less than 1 cm, (2) between 1 cm and 2 cm, (3) between 2 cm and 4 cm, and (4) larger than 4 cm.

The patients' gender, age, extrathyroidal invasion, multifocality, tumor size, and T stage were the inclusion criteria for the study. Indicators such as lymph node metastasis, the extent of surgical dissection, pathological subtype, and gene mutation were the exclusion criteria. The ratio of males to females was about 1 : 3, and the median age was 43 years.

### 2.2. The Nodal Staging Score (NSS) Statistical Model

In this study, the beta-binomial distribution was used to establish a nodal staging score (NSS) model [11, 12]. The beta-binomial distribution is a compound distribution that assumes that the parameter *p* in the binomial distribution is a random variable and obeys the *β* distribution [13]. Thus, this method was adopted.

### 2.3. Statistical Analysis

The NSS model was built through R 3.3.2 (R Foundation for Statistical Computing, Vienna, Austria). The parameters *α* and *β* to be estimated in the beta-binomial distribution were calculated by the maximum likelihood estimation method using the VGAM package. Other data were statistically analyzed using SPSS version 22.0 (SPSS Inc., Chicago, IL, USA). Comparisons were made using the *χ*^2^ test for categorical variables and the *t*-test for continuous variables in the basic patient characteristics. It was believed that there was a statistically significant differences where *p* is less than 0.05.

## 3. Results

### 3.1. Basic Information of the Patients

A total of 12,431 patients with PTC in the SEER database from 2000 to 2005 met the inclusion/exclusion criteria and were included in this study. Six clinicopathological factors that may affect lymph node metastasis and the negative predictive value of lymph nodes were grouped and analyzed. In addition, 4,866 (39.1%) patients received total thyroidectomy and 2,450 (19.7%) underwent lateral cervical lymphadenectomy. Among the 12,431 patients, 5,959 (47.9%) were positive for lymph node metastasis, and 6,472 (52.1%) were confirmed negative, which shows a significant difference in the proportion of patients with all study factors between these two groups (*p* > 0.05). The basic information about the patients and the number of examined lymph nodes are shown in Table 1.

### 3.2. Calculation of the Probability of False-Negative Lymph Node Metastasis

**Figure 1:**
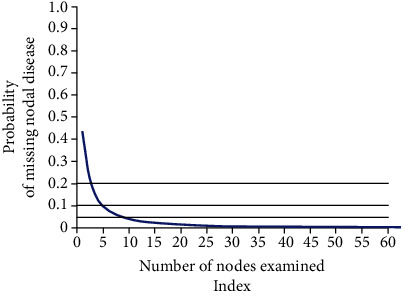
The relationship between the number of lymph nodes examined and the probability of false-negative lymph node metastasis.

A total of 5,959 patients with PTC had at least one lymph node metastasis. The beta-binomial distribution model was used to analyze the distribution of lymph node metastasis rates in patients, and the parameters to be estimated were *α* = 1.51 and *β* = 1.15. As shown in Figure 1, when one, three, five, or eight nodes are examined, the false-negative probabilities of metastatic lymph nodes in PTC patients are 42.2%, 18.3%, 9.3%, and 5.6%, respectively. When more than eight lymph nodes are examined, the probability of false-negative lymph node metastasis is less than 5%. The calculation of the false-negative probability of lymph node metastasis is determined only by the total number of lymph nodes examined, and there is no correlation between tumor pathology and patient factors.

### 3.3. Evaluation of the Incidence of Lymph Node Metastasis

The incidence of lymph node metastasis should be evaluated based on the total number of examined lymph nodes and possible factors. The incidence of lymph node metastasis was assessed in all 12,431 PTC patients (Table 2). The corrected incidence of lymph node metastasis was higher than that of direct calculation, regardless of which factors affected lymph node metastasis.

### 3.4. Assessment of Occult Lymph Node Metastasis Risks

**Figure 2:**
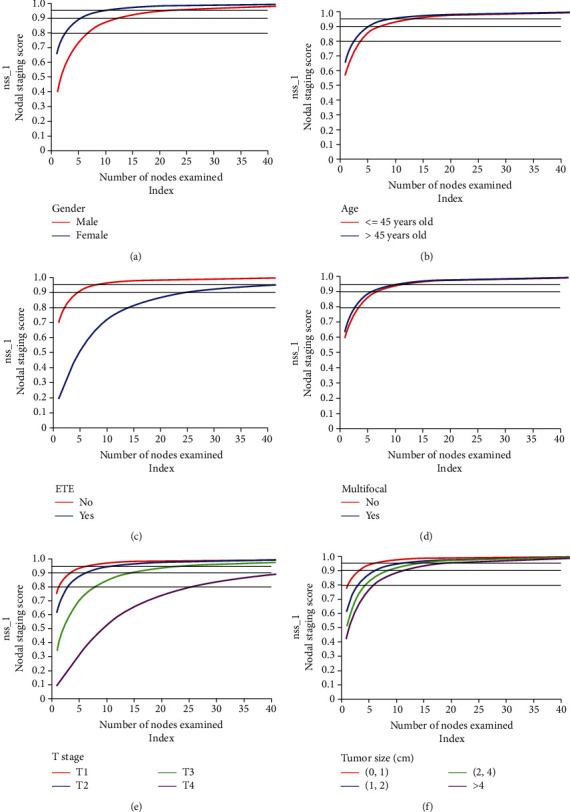
NSS results of lymph node metastasis corresponding to different clinicopathological factors. (a) Patient's gender; (b) patient's age (age 45 and under 45; older than 45 years old); (c) extrathyroidal invasion; (d) multifocal tumor; (e) T stages in line with the 7th edition of AJCC staging (T1, T2, T3, and T4); (f) tumor size (less than 1 cm; between 1 cm and 2 cm; between 2 cm and 4 cm; larger than 4 cm).

Based on the previous calculation of the possibility of false-negative lymph node metastasis and the adjusted incidence of lymph node metastasis, we have further assessed the risk of occult lymph node metastasis in PTC patients after receiving lymphadenectomy, i.e., the nodal staging score (NSS) model. The gender, age, extrathyroidal invasion, multifocality, tumor size, and T stage of patients were included in the derivation process, respectively, and the corresponding NSS results are shown in Figure 2. Regardless of the influencing factors of lymph node metastasis, these results suggest the number of lymph nodes to be removed to achieve a certain predictive value (Table 3) and illustrate the risk of residual occult lymph node metastasis after a certain number of lymph nodes are removed (Table 4).

To achieve a negative predictive value of more than 90%, 12 and 6 lymph nodes were examined in males and females, respectively; for PTC patients who were at age of 45 or less or more, 8 and 6 lymph nodes were examined, respectively; twenty-two and five lymph nodes were examined in individuals with and without extrathyroidal invasion, respectively; patients with and without multifocal tumors, examination of seven and six lymph nodes was required, respectively; for patients whose tumor sizes were> 1 cm, 1-2 cm, 2-4 cm, and> 4 cm, four, seven, nine, and thirteen lymph nodes were examined, respectively; and for patients in T1, T2, and T3 stages, four, six, and fifteen lymph nodes were examined, respectively.

### 3.5. Impact of NSS Assessment on Patient Survival

The NSS results calculated based on the affecting factors of lymph node metastasis were divided into four groups following their respective quartiles to evaluate the impact of the risk of occult lymph node metastasis on the prognosis of patients, and the overall survival (OS) among the four groups was compared by Log-rank method. The results showed that there were significant differences in the OS of PTC patients among the four groups in terms of gender, age, extrathyroidal invasion, tumor multifocality, tumor size, and T stage (*p* is less than 0.05), indicating that cervical lymph node metastasis would have an impact on the prognosis of patients, and the NSS model can better predict the cervical lymph node metastasis.

## 4. Discussion

Cervical lymph node metastasis often occurs in PTC, and cervical lymph node recurrence is a common type of postoperative recurrence in PTC [1, 3, 14]. Therefore, both proper assessments of lymph node metastasis and appropriate treatment are very important for improving the disease-free survival of patients. The inaccuracy of preoperative lymph node metastasis assessment will affect the formulation of treatment plans. Occult central lymph node metastases are easily missed due to the occlusion of the thyroid lobes during preoperative ultrasonography, and 66% of the central lymph node metastases are smaller than 5 mm [15]. Therefore, the Guideline for Diagnosis and Treatment of Thyroid Nodule and Differentiated Thyroid Cancer (2012 edition) of China recommends that at least an ipsilateral lobule and isthmus resection and ipsilateral central lymph node dissection should be operated on patients with differentiated thyroid cancer, but there is still a lack of opinions on the number of lymph nodes to be dissected [16]. Furthermore, lymph node metastasis in PTC patients is one of the indicators to be evaluated for postoperative radioactive iodine therapy. It is particularly important to correctly assess the patient's lymph node metastasis considering the possible side effects of radioactive iodine therapy [4]. However, for patients with no evidence of lymph node metastasis by preoperative ultrasound and Tg evaluation, who had received thyroid and/or lymph node dissection, and were followed up regularly with ultrasound and Tg after surgery, 77% still showed abnormalities within one year after the surgery [7], which may be related to postoperative occult lymph node metastasis. Preventive central lymph node dissection is widely adopted recently, while lateral cervical lymph node dissection is only carried out upon high suspicion/confirmation of metastasis through pathological biopsy [17]. For PTC patients, we would study how many lymph nodes will be removed for examination during lymph node dissection is enough to determine metastasis, and determine that patients with negative lymph node metastases are still at risk for occult metastases based on postoperative pathology reports. The lymph node dissection for thyroid cancer still lacks an “indicator” like “the sentinel lymph node” for breast cancer [14] due to the advanced cervical lymphatic circulation system. The thoroughness of surgery is still needed in the treatment of the occult metastatic lymph nodes. Therefore, this study is aimed at exploring the NSS model of cervical lymph node metastasis in PTC patients with the help of a large sample database and statistical analysis model. Individualized therapy is increasingly important in oncology treatment, and the NSS model can provide an individualized assessment of the risk of occult lymph node metastasis in patients with PTC.

Occult lymph node metastases are lymph node metastases that are not detected by clinical examination and procedure and are later confirmed by pathological examination, with a reported incidence of up to 50% in PTC. The current preoperative examination of lymph node metastases in patients with PTC is primarily based on ultrasonography [2, 14]. The inaccuracy of preoperative lymph node metastasis assessment will affect the formulation of the treatment plan. Because occult lymph node metastases are commonly observed in the central area, many physicians are in support of prophylactic central lymph node dissection, but there is a lack of opinions on the number of lymph nodes removed.

The NSS model is mainly used to assess the negative predictive value of lymph nodes in patients through a comprehensive analysis of the number, metastasis, and factors affecting the lymph nodes, and therefore can be used in assessments of the patient's condition and guide for relevant treatment strategies. Robinson et al. have reported the establishment of an assessment system for lymph node metastasis in patients with colon cancer using the NSS model, and Gonen et al. later preliminarily explored the application of the NSS model in PTC patients [11, 12].

Following are the main conditions for establishment of this model: (1) no false-positive metastasis in lymph nodes; (2) all lymph nodes have the same possibility of metastasis; and (3) the examinations are equally sensitive to true positives and false negatives. The adjusted incidence is higher than the incidence of lymph node metastasis calculated which evaluates the possibility of lymph nodes.

Based on the analysis of the survival data in patients with PTC, we have found that cervical lymph node metastasis can affect the prognosis of patients, and the NSS can better predict cervical lymph node metastasis. Besides, the NSS calculation was also carried out on age and extrathyroidal invasion. For any patient, however, the impact of each factor on lymph node metastasis was not completely independent. Moreover, one of the premises of this study is that each lymph node has the same probability of metastasis. But for PTC, the possibility of lymph node metastasis varies in different areas, with the most common metastasis in the central area, followed by the metastasis in the cervical area, while the incidence of mediastinal metastasis is the lowest [2].

## 5. Conclusion

This paper provides an intuitive and quantitative assessment for the predictive value of a negative lymph node examination based on factors such as gender, age, extrathyroidal invasion, tumor multifocality, tumor size, and T stage, and establishes a lymph node assessment system, which is mainly used for the assessment of the risk of occult lymph node metastasis and offers guidance on patient treatment strategies.

## Figures and Tables

**Table 1:** Basic information of the patients and the number of examined lymph nodes.

	The number of patients (%)^&^			*p* value	The number of examined lymph nodes	
	Total (*n* = 12431)	LN + (*n* = 5959)	LN − (*n* = 6472)		The median	IQR^#^
Gender				<0.05		
Male	2886(23.2)	1825(30.6)	1061(16.4)		3	1(-10)
Female	9545(76.8)	4134(69.4)	5411(83.6)		2	1(-6)
Age (years old)				<0.05		
≤45	7127(57.3)	3706(62.2)	3421(52.9)		3	1(-7)
>45	5304(42.7)	2253(38.7)	3051(47.1)		2	1(-5)
Extrathyroidal invasion				<0.05		
Negative	9496(76.4)	3752(63.0)	5744(88.8)		2	1(-5)
Positive	2935(23.6)	2207(37.0)	728(11.2)		4	2(-12)
Multifocal				<0.05		
No	8431(67.8)	4157(69.8)	4274(66.0)		3	1(-7)
Yes	4000(32.2)	1802(30.2)	2198(34.0)		2	2(-3)
Tumor size^∗^				<0.05		
≤1 cm	3517(28.3)	1137(19.1)	2380(36.8)		2	1(-4)
1–2 cm	3803(30.6)	1817(30.5)	1986(30.7)		2	1(-6)
2–4 cm	3190(25.7)	1780(29.9)	1410(21.8)		3	1(-7)
> 4 cm	1062(8.5)	683(11.5)	379(5.9)		4	1(-12)
T stage				<0.05		
T1	6121(49.2)	2072(34.8)	4049(62.6)		2	1(-5)
T2	2237(18.0)	1060(17.8)	1177(18.2)		2	1(-6)
T3	3146(25.3)	2094(35.1)	1052(16.3)		3	1(-10)
T4	927(7.5)	733(12.3)	194(3.0)		4	2(-14)

Note: ^∗^Tumor size data were not available for 859 (6.9%) of these patients; #IQR refers to interquartile range; &: In the number of patients, LN+ indicates patients with positive lymph node metastasis, and LN- indicates patients with negative lymph node metastasis.

**Table 2:** Incidence of lymph node metastasis in patients with PTC and its adjustment results.

	Incidence of lymph node metastasis	
	Incidence of the sample (%)	The adjusted incidence (%) ^#^
Gender		
Male	63.2	77.6
Female	43.3	53.8
Age		
≤45	52.0	63.2
>45	42.5	53.8
Extrathyroidal invasion		
Negative	39.5	49.5
Positive	75.2	90.5
Multifocal		
No	49.3	60.6
Yes	45.1	56.3
Tumor size		
≤1 cm	32.3	40.2
1-2 cm	47.8	59.1
2-4 cm	55.8	68.8
> 4 cm	64.3	76.0
T stage		
T1	33.9	42.4
T2	47.4	58.3
T3	66.6	81.5
T4	79.1	95.7

Note: #: In consideration of the possibility of false-negative lymph node metastasis, the incidence of lymph node metastasis calculated directly was revised.

**Table 3:** NSS corresponds to the number of lymph nodes examined.

NSS	The number of lymph nodes examined			
	80%	85%	90%	95%
Gender				
Male	7	9	12	21
Female	3	4	6	10
Age				
≤45	4	5	8	13
>45	3	4	6	10
Extrathyroidal invasion				
Negative	2	3	5	9
Positive	14	18	22	40
Multifocal				
No	4	5	7	12
Yes	3	4	6	10
Tumor size				
≤1 cm	2	2	4	6
1-2 cm	3	5	7	11
2-4 cm	5	6	9	15
> 4 cm	4	8	13	20
T stage				
T1	2	3	4	7
T2	3	5	6	11
T3	3	11	15	25
T4	25	32	—	—

**Table 4:** Number of lymph nodes examined corresponding to NSS values.

The number of lymph nodes examined	NSS (%)					
	1	5	10	15	20	25
Gender						
Male	40.0	74.7	87.6	92.4	94.8	96.0
Female	66.5	89.8	95.5	97.3	98.2	98.7
Age						
≤45	57.4	85.6	93.4	96.1	97.4	98.1
>45	66.5	89.8	95.5	97.3	98.2	98.6
Extrathyroidal invasion						
Negative	70.2	91.3	96.1	97.7	98.5	98.9
Positive	19.4	51.7	71.9	81.6	88.3	91.8
Multifocal						
No	60.0	86.9	94.1	96.5	97.6	98.3
Yes	64.2	88.8	95.0	97.1	98.0	98.5
Tumor size						
≤1 cm	77.4	93.9	97.4	98.5	99.0	99.2
1-2 cm	61.5	87.7	94.5	96.7	97.8	98.3
2-4 cm	51.2	82.3	91.8	95.1	96.9	97.4
> 4 cm	42.1	76.4	88.6	93.1	95.0	95.9
T stage						
T1	75.9	93.3	97.1	98.3	98.9	99.2
T2	62.3	88.0	94.6	96.8	97.5	98.0
T3	34.4	69.9	84.7	90.6	93.3	95.0
T4	9.4	31.6	49.0	65.6	73.3	80.0

## Data Availability

The data used to support the findings of this study are included within the article.

## References

[1] Hundahl S. A., Fleming I. D., Fremgen A. M., Menck H. R. (1998). A National Cancer Data Base report on 53,856 cases of thyroid carcinoma treated in the U.S., 1985-1995. *Cancer: Interdisciplinary International Journal of the American Cancer Society*.

[2] Grebe S. K., Hay I. D. (1996). Thyroid cancer nodal metastases: biologic significance and therapeutic Considerations. *Surgical Oncology Clinics of North America*.

[3] Scheumann G. F., Gimm O., Wegener G., Hundeshagen H., Dralle H. (1994). Prognostic significance and surgical management of locoregional lymph node metastases in papillary thyroid cancer. *World Journal of Surgery*.

[4] Haugen B. R., Alexander E. K., Bible K. C. (2016). 2015 American Thyroid Association management guidelines for adult patients with thyroid nodules and differentiated thyroid Cancer: the American Thyroid Association guidelines task force on thyroid nodules and differentiated thyroid Cancer. *Thyroid*.

[5] Berger M. F., Levin J. Z., Vijayendran K. (2010). Integrative analysis of the melanoma transcriptome. *Genome Research*.

[6] Oh H. S., Ahn J. H., Song E. (2019). Individualized follow-up strategy for patients with an indeterminate response to initial therapy for papillary thyroid carcinoma. *Thyroid*.

[7] Bates M. F., Lamas M. R., Randle R. W. (2018). Back so soon? Is early recurrence of papillary thyroid cancer really just persistent disease?. *Surgery*.

[8] Jung J. H., Kim C. Y., Son S. H. (2015). Preoperative prediction of cervical lymph node metastasis using primary tumor SUVmax on 18F-FDG PET/CT in patients with papillary thyroid carcinoma. *PLoS One*.

[9] Hartl D. M., Leboulleux S., Al Ghuzlan A. (2012). Optimization of staging of the neck with prophylactic central and lateral neck dissection for papillary thyroid carcinoma. *Annals of Surgery, (2012)*.

[10] (2019). SEER Cancer Statistics Review, [National Cancer Institute]. https://seer.cancer.

[11] Robinson T. J., Thomas S., Dinan M. A., Roman S., Sosa J. A., Hyslop T. (2016). How many lymph nodes are enough? Assessing the adequacy of lymph node yield for papillary thyroid cancer. *Journal of Clinical Oncology*.

[12] Gonen M., Schrag D., Weiser M. R. (2009). Nodal staging score: a tool to assess adequate staging of node-negative colon cancer. *Journal of Clinical Oncology*.

[13] Chen G., Yang S. Q. *β*-binomial distribution and its medical application. *Chinese Journal of Health Statistics*.

[14] Sherman S. I. (2003). Thyroid carcinoma. *The Lancet*.

[15] Vergez S., Sarini J., Percodani J., Serrano E., Caron P. (2010). Lymph node management in clinically node-negative patients with papillary thyroid carcinoma. *European Journal of Surgical Oncology*.

[16] Gao M. (2012). Guideline for diagnosis and treatment of thyroid nodular and differentiated thyroid carcinoma. *Chinese Journal of Clinical Oncology*.

[17] Machens A., Hauptmann S., Dralle H. (2009). Lymph node dissection in the lateral neck for completion in central node-positive papillary thyroid cancer. *Surgery*.

[18] Xu Z. G., Liu S. Y. (2017). Expert Consensus on Cervical Lymph Node Dissection in Differentiated Thyroid Carcinoma (2017 Edition). *Chinese Journal of Practical Surgery*.

[19] Alroy I., Yarden Y. (1997). The ErbB signaling network in embryogenesis and oncogenesis: signal diversification through combinatorial ligand-receptor interactions. *FEBS Letters*.

[20] Ferlay J., Colombet M., Soerjomataram I. (2019). Estimating the global cancer incidence and mortality in 2018: GLOBOCAN sources and methods. *International Journal of Cancer*.

